# Factors Associated with Increased Intraocular Pressure in Type 2 Diabetes Patients

**DOI:** 10.3390/jcm13030676

**Published:** 2024-01-24

**Authors:** Adina Braha, Amanda Simion, Romulus Timar, Bogdan Timar

**Affiliations:** 1 Department of Second Internal Medicine—Diabetes, Nutrition, Metabolic Diseases, and Systemic Rheumatology, “Victor Babeș” University of Medicine and Pharmacy, 300041 Timisoara, Romania; braha.adina@umft.ro (A.B.); timar.romulus@umft.ro (R.T.); bogdan.timar@umft.ro (B.T.); 2 Department of Diabetes, Nutrition and Metabolic Diseases Clinic, “Pius Brînzeu” Emergency Clinical County University Hospital, 300723 Timisoara, Romania; 3 Vista Vision Ophthalmology Clinic, 300367 Timisoara, Romania

**Keywords:** intraocular pressure, type 2 diabetes, diabetic eye, predictive factors, hemoglobin A1c, diabetes duration

## Abstract

Background: Over one-third of patients living with diabetes will develop ocular disease during their lifetime. The present study analyzes the association between metabolic and anthropometric markers, associated comorbidities, and intraocular pressure (IOP) in patients with type 2 diabetes mellitus (DM). Methods: The retrospective study included 87 adult patients with type 2 DM who underwent routine eye examinations and blood/urine tests. Results: 67.9% of the patients had an IOP > 14.5 mmHg and only 41.3% had an HbA1c < 7%. In a multivariate regression analysis, the mean IOP was associated with diabetes duration in subjects with a disease duration < 15 years and an HbA1c < 7% (adjusted R^2^ = 0.29, *p* = 0.008). Also, patients with shorter diabetes duration and optimal glucose control had a lower mean IOP than patients with a higher HbA1c (mean IOP 15.1 mmHG vs. 18.9 mmHg, *p* = 0.04). The patient’s age, anthropometric or metabolic markers, associated comorbidities like hypertension (HTN) or retinal angiosclerosis, and diabetes therapies were not associated with IOP in this study. Conclusion: Diabetes progression is directly associated with increased IOP. Avoiding clinical inertia and optimizing glycemic control could prevent or delay the increase of IOP. Routine eye examination should include measuring IOP, not only diabetic retinopathy screening.

## 1. Introduction

The continuing global increase in DM prevalence is responsible for 6.7 million deaths. Chronic hyperglycemia is causing degenerative complications in both micro and macrovascular territories. During their lifetime, up to one-third of people living with DM will develop cardiovascular disease (CVD) [1], up to 40% of them will suffer from chronic kidney disease (CKD) [2], and over one-third of them will develop some form of eye disease and, eventually, loss of vision or even blindness [3]. High IOP [4,5,6] and glaucoma [7] are included among the many ocular complications related to DM.

IOP is the pressure inside the eye, while intracranial pressure is the pressure inside the skull. Both IOP and intracranial pressure can affect the optic nerve. Glaucoma is a chronic condition that leads to vision loss and blindness by damaging the optic nerve. One of the mechanisms underlying IOP and glaucoma is the translaminar pressure gradient, which can cause stress and strain on the lamina cribosa, which may impair the blood flow and axonal transport of the optic nerve fibers, resulting in cell death and nerve damage [8]. Another underlying mechanism is nitric oxide (NO), which produces aqueous humor, the fluid that fills the front part of the eye and maintains IOP. NO also affects the blood flow to the optic nerve and the retina, the light-sensitive tissue at the back of the eye. Impaired NO signaling can contribute to glaucoma development through vascular and mechanical mechanisms, and increased IOP appears to play a more significant role [9].

These mechanisms link IOP and glaucoma, but other factors may influence the disease process, such as genetics, inflammation, autoimmunity, and ischemia [10]. The connection between intraocular pressure (IOP), glaucoma, and diabetes is not fully understood. DM may increase the risk of glaucoma by affecting the blood vessels in the eye, causing them to become more permeable, leaky, or constricted. This can lead to changes in IOP, optic nerve damage, or impaired blood flow to the retina [11,12]. Another possible mechanism of the association of hyperglycemia with increased IOP is the relationship between the increased expression of extracellular matrix components, including fibronectin, and the increased IOP in chronic hyperglycemia. For example, fibronectin is related to the pathogenesis of POAG and increased IOP [13,14]. The expression of fibronectin is increased under high glucose in trabecular meshwork cells, which is related to increased trabecular meshwork outflow resistance [15,16]. Moreover, hyperglycemia is associated with increased IOP in patients with DM [17], independent of central corneal thickness [7].

Today, the American Diabetes Association Standards of Care guidelines recommend screening for diabetic retinopathy at the diagnosis of DM and, if normal, at least every 1–2 years. For reducing the risk or slowing the progression of diabetic retinopathy, optimizing glucose control, blood pressure, and lipids is recommended. A glycated hemoglobin A1c below 7% without hypoglycemia or weight gain, respectively, and treatment with sodium-glucose co-transporters-2 (SGLT-2) inhibitors and glucagon-like peptide-1 (GLP-1) receptor agonists are strongly recommended in patients living with type 2 DM for preventing or slowing the progression of CVD or CKD.

However, evidence about the effects of this glycemic target or antidiabetic molecules on IOP is lacking. The present study aims to address this gap and investigate the association between IOP and glycemic control, metabolic factors, and different treatment regimens in a Romanian population with type 2 DM.

## 2. Materials and Methods

We analyzed retrospective data on the Diabetes Center of Emergency County Hospital Pius Brinzeu Timisoara, Romania, searching for patients with DM who received a medical request note for a routine eye examination for screening diabetic retinopathy during August–November 2023 and identified 172 patients. The informed consent was waived due to the retrospective study design. The present research is part of an ongoing study evaluating the effects of different treatment regimens on metabolism, nutritional status, and behavior in type 2 DM patients. It was approved by the Ethical Committee of the Emergency County Hospital Pius Brinzeu Timisoara, Romania (406/29 September 2023), and was conducted according to the Helsinki Declaration (version 2013), respecting the confidentiality of patients’ personal data, according to General Data Protection Regulation (GDPR) Compliance.

The inclusion criteria were type 2 DM, age > 18 years, complete data about eye examination results, and blood/urine tests performed 90 days before or after the eye examination. Type 1 DM, previously diagnosed eye disease, or missing data excluded patients from the study. Most of the excluded patients had missing data about the eye examination.

Data about patient anthropometrics, metabolic characteristics, medical history, and diabetic therapy were obtained from the patient’s medical file. The associated comorbidities were previously diagnosed and documented in the patient’s medical file.

The study’s main outcome was increased IOP measured by Goldmann Tonometry with a threshold of 14.5 mmHg based on a mouse model study developed for investigating potential therapies for glaucoma [18]. IOP was measured for both eyes, and mean IOP represents the average IOP of the left and right eyes. Other variables from the eye examination, like diabetic retinopathy of different grades, maculopathy, cataract, glaucoma, and retinal angiosclerosis, were analyzed.

### Statistical Analysis

The data were statistically analyzed with MedCalc^®^ Statistical Software version 22.016 (MedCalc Software Ltd., Ostend, Belgium, 2023) and GNU pspp 1.4.1. To meet the desired statistical constraints for the study outcome, we calculated the sample size based on a 95% confidence level with a margin of error of 17% (18), which resulted in a minimum of 34 study subjects. Continuous numerical variables were tested for normal distribution with the Shapiro–Wilk test. Normal-distributed variables were presented as mean and standard deviation, while non-parametric variables were presented as median and minimum-maximum values. The qualitative/nominal variables are the number of individuals in the class and the percentage of the total subgroup. To evaluate the differences between the indicators of the central tendency between the groups, we used *t*-student tests (paired/unpaired) to compare the arithmetic means of the parametric variables between two groups, Mann–Whitney-U tests, for comparing two medians. We used chi-square tests to compare the statistical significance of the differences between the two proportions. The strength of the associations between the numerical variables analyzed was evaluated with the help of bivariate regressions. We calculated the coefficient of determination (R^2^) to verify in what proportion the variation of the independent variable generates the variation of the dependent variable. In order to verify the statistical significance of the analyzed correlations and, consequently, the possibility of generalizing the association in the population, we used the distribution test of t values, which takes into account the value of the correlation coefficient and the size of the studied sample. In the study, we calculated the 95% confidence interval and considered a *p*-value of less than 0.05 as significant for the statistical analyses.

## 3. Results

The final analysis comprised 87 patients, most of whom were women 46/87 (52.8%). The mean age was 63.5 years, with no differences between genders. The median duration of diabetes was 9 years, similar in men and women. The majority of patients were overweight or with different grades of abdominal obesity, as presented in Table 1. The lipid profile was modified in both genders but with a significantly higher HDLc in women. Men had significantly higher serum creatinine and body weight (*p* = 0.008, respectively 0.0009), but the eGFR and BMI were similar both in men and women. There were no differences concerning liver function, regardless of gender. The general characteristics of the included patients, depending on gender, are presented in Table 1.

The HbA1c ranged from 5% to 12% in the studied patients, with no significant differences among men or women. The median HbA1c was 6.8%. Among these patients, 36/87 (41.3%) achieved the recommended glycemic target below 7%. Patients were treated by their diabetologist according to the standard of care [19] depending on their comorbidities and personalized treatment targets, with different molecules and combinations, as presented in Table 2. A high percentage of the patients were treated also for HTN (76.3%), coronary heart disease (CHD) (14.9%), and CKD (17.3%). A lower percentage of the patients developed chronic degenerative complications due to diabetes: 10.6% retinopathy, 8% sensitive neuropathy, 5.3% peripheral arterial disease, and 5.4% stroke. Most of the diabetic retinopathy was nonproliferative (6.9%), as presented in Table 2. However, cataracts were more prevalent among these patients, similarly in both eyes (46% and 44%, respectively). In addition, 13.7% (12/87) of patients presented an IOP above 21 mmHg, and 8.3% of the studied subjects were diagnosed with glaucoma. A total of 59.8% of the patients had retinal angiosclerosis, and only 4.6% of them presented with myopia. The associated comorbidities and treatment regimens are presented in Table 2.

In the present study, 60/87 (68.9%) of the patients presented an IOP ≥ 14.5 mmHg (*p* = 0.002). There were no differences in age, diabetes duration, constitutional type (body weight, BMI, waist), glycemic control (HbA1c, fasting glycemia), lipid profile (TC, TG, HDLc, LDLc), or renal function. Among the patients with an IOP ≥ 14.5 mmHg, 19 (33.3%) were treated with statins, compared to the other group, where 43.3% were on a statin (*p* = 0.3). The group with higher IOP had lower mean values in ALP (*p* = 0.03), higher median values in GGT (*p* = 0.007), and mean TBil (*p* = 0.02) but no significant differences in liver transaminases (AST, ALT). Table 3 compares anthropometric and metabolic variables according to the outcome threshold for IOP.

**Figure 1:**
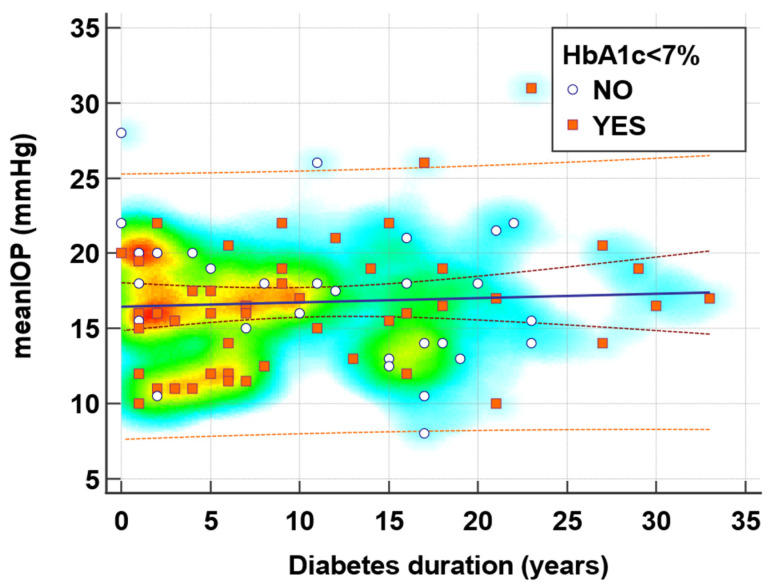
Regression analysis of mean intraocular pressure and diabetes duration in type 2 diabetes patients who achieved the target of HbA1c < 7% compared to those with uncontrolled glycemia.

**Figure 2:**
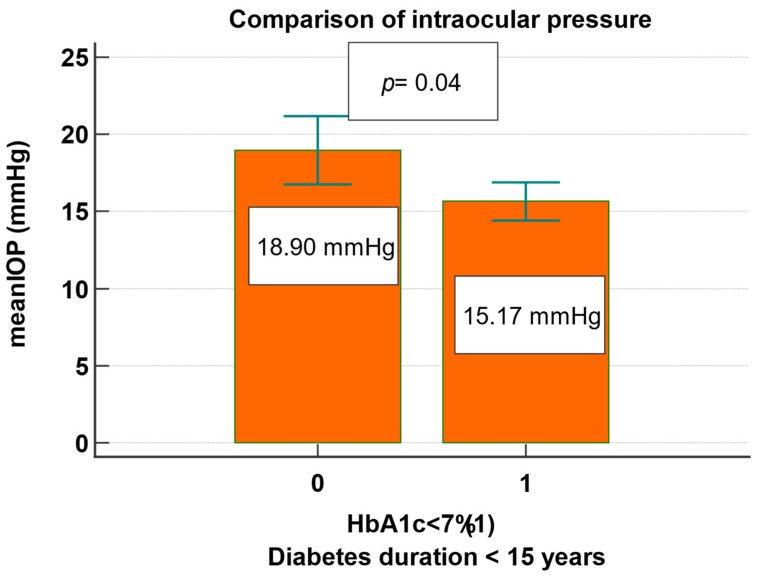
Comparison of mean intraocular pressure in type 2 diabetes patients with a disease duration below 15 years and glucose control.

The mean IOP was associated with diabetes duration in a regression subanalysis that included 41 (47.1%) of the studied subjects who had a diabetes duration of < 15 years and HbA1c < 7% (Figure 1). In this subgroup, 26 (63.4%) patients had a significant optimal glucose control and a lower mean IOP of 15.1 mmHg, compared to patients with a higher HbA1c who presented a higher mean IOP of 18.9 mmHg, *p* = 0.04 for both (Figure 2).

According to this regression equation (Table 4), an increase in the standard deviation of the diabetes duration is expected to increase the IOP by 0.52 mmHg in type 2 DM patients with an HbA1c < 7% and a diabetes duration < 15 years (*p* = 0.004, adjusted R^2^ = 0.29). The results indicate that the diabetes progression appears to be directly associated with IOP. The patient’s age, anthropometric or metabolic markers, associated comorbidities like HTN or retinal angiosclerosis, and diabetes therapies were not associated with IOP in this study.

## 4. Discussion

This Romanian population-based retrospective research assessed the association of glycemic control (HbA1c and glycemia), lipid profile, liver and kidney function, and anthropometric and medical characteristics with IOP in patients with type 2 DM without previously known ocular disease.

Recent diabetes guidelines focus mainly on treating diabetes with SGLT2 inhibitors and GLP-1 RA as an add-on to metformin and lifestyle changes but based on associated comorbidities like heart failure, atherosclerosis, and chronic kidney disease, aiming to increase the percentages of patients who achieve an HbA1c < 7% for reducing chronic comorbidities [20]. However, there is a gap regarding glycemic control and different ocular diseases. Indeed, achieving an HbA1c of 6.6–7% results in a significant reduction of 23% in the requirement of retinal photocoagulation [21].

In the present study, we included patients treated according to the latest standard of care [20] (Table 2), and about 41% achieved the desired HbA1c < 7%. Among them, we identified different grades of retinopathy in about 10% of the cases and glaucoma in about 8% of the subjects. Although a significant percentage of the patients achieved glycemic control, the prevalence of glaucoma in our study was increased, compared to a recent meta-analysis where the prevalence of primary open-angle glaucoma ranged between 2.8% and 3.6% [11,22]. Moreover, the results showed a high percentage—67.9%—of patients with IOP > 14.5 mmHg. Previous research has proved that up to 80% of diabetes patients present with ocular hypertension compared with patients without any form of diabetes, even after adjusting for central corneal thickness [23]. A recent meta-analysis showed that patients with diabetes are 1.48 and 1.52 times more likely to develop glaucoma and ocular hypertension, respectively [24].

In our study, in patients with less than 15 years’ duration of diabetes, HbA1c was associated with increased IOP, suggesting that poor glycemic control is a risk factor for increased IOP, and many of these patients could eventually develop glaucoma [25]. In other studies, higher IOP was associated with hyperglycemia [26,27,28]. However, recent findings in the literature are controversial, suggesting a U-shaped relation between HbA1c and IOP; very poor or very strict glycemic control may increase IOP [21]. It is speculated that strict glycemic control could cause endothelial dysfunction, altered ocular blood, and aqueous humor flow [21]. In a large Korean study, HbA1c was inversely correlated with IOP in patients with diabetic retinopathy compared to those without retinopathy, suggesting that hyperglycemia could increase the oxidative stress and the levels of vascular endothelial growth factor, thus altering the outflow of the aqueous humor resulting in reduced IOP [29]. Optimal glucose control may not be enough for all patients with diabetes when considering reducing IOP, and one possible explanation could be the challenge of achieving and maintaining glycemic control over time. The present findings did not connect any diabetic medication to IOP. There are only a few small studies that have investigated the impact of different classes of antidiabetic drugs on IOP or glaucoma in patients with diabetes. Metformin has some benefits in preventing open-angle glaucoma [30] due to several mechanisms beyond glycemic control [31]. There are also other classes of molecules with some evidence of being beneficial to IOP. For example, SGLT2 inhibitors may improve ocular outcomes, but due to the limited sample size, no conclusions can be drawn [32]. The GLP-1 receptor agonists have promising results in studies with glaucoma [33]. However, the mechanisms of these two classes in treating ocular pressure are not well understood and require further investigation.

Of course, other factors could influence IOP beyond glycemic control, such as smoking, medication, dyslipidemia, hypertension, obesity, genetics, or other ocular disorders [23]. In our findings, 59.8% of the patients presented retinal angioslerosis, which was not associated with our outcome in the regression analysis. However, blood pressure control and therapy data were unavailable for analysis. The IOP is a variable with a linear trend and was previously analyzed in several other research studies. In a Tehran Geriatric Eye study report, the researchers found that an IOP ranging from 14 to 17 mm Hg was associated with older age and high systolic blood pressure [34]. A meta-analysis of European cohort studies reported an inverted U-shape trend, indicating that IOP increased until the age of 60 and then decreased again in older people [35]. However, in our study, the IOP was not associated with age but with the diabetes duration in patients with an HbA1c < 7% and less than 15 years of disease.

Also, elevated blood pressure is associated with elevated IOP, leading to an increased risk of glaucoma. However, excessive blood pressure lowering in patients suffering from glaucoma may drop the ocular perfusion pressure and provoke subsequent ischemic injury. The relationship between IOP, ocular perfusion, and blood pressure suggests that blood pressure and glaucoma progression have a U-shaped curve [36]. Although not all patients have symptoms, there is a risk of choroidal expansion and hypotony maculopathy [37]. Therefore, the optimal IOP in patients with type 2 DM needs further investigation.

In some studies, an altered lipid profile was associated with increased IOP [26,38,39,40,41] but not glaucoma [41]. In contrast, in other studies, increased consumption of n-3 or n-6 polyunsaturated fat increased the risk of primary open-angle glaucoma [42]. The exact mechanisms for the relationship between dyslipidemia and IOP are not fully understood. One possible explanation is that increased lipid levels could increase the episcleral venous pressure and blood viscosity, thus reducing the outflow facility [43]. Second, high levels of lipids could alter the meibomian glands that regulate the lipid secretion within the eyelids, leading to dry eye disease, which is associated with increased IOP [44]. In our analysis, no correlation between lipids and IOP was found. However, patients with elevated IOP presented significantly higher GGT and Tbil values and lower ALP values. In a similar study, there was a linear relationship between increased IOP and the severity of fatty liver [45] in patients with chronic alcohol consumption or high-lipid or high-fructose diets, respectively, in patients with nonalcoholic fatty liver disease [46]. The authors explained the findings by stating that altered gut permeability is responsible for ectopic immune stimulation in the liver [39] with the secretion of proinflammatory cytokines that develop atherosclerosis. Atherosclerosis contributes to the dysfunction of IOP and blood flow [47]. An interesting observation in our study is the lower values of ALP in patients with higher values of IOP. The ALP is an enzyme found in many body parts, including the liver and bones. The exact relationship between ALP and IOP is not fully understood. It may vary depending on the underlying conditions [48,49], like liver dysfunction or bone disorders, e.g., osteoporosis, that can alter calcemia and phosphatemia [50], which are also involved in eye function.

However, the present study’s design is limited in showing a causal relationship between analyzed factors or potential mechanisms underlying these relationships. Also, we did not measure the central corneal thickness that could influence the IOP. The results of this study should be interpreted with caution because the duration of type 2 DM cannot be determined accurately. The relatively small number of analyzed patients did not allow powerful subanalysis for different antidiabetic molecules that patients were treated with to establish a possible connection between the potential mechanisms of the drugs and the dynamics of IOP. Moreover, several factors, like chronic consumption of alcohol or serum values of calcium and phosphorus, were missing in the present study. Further studies are needed to confirm and explain these findings.

## 5. Conclusions

Therefore, the relationship between ocular hypertension and metabolic control is complex and variable, and intense glycemic control may be beneficial for patients with diabetes before the disease progresses. Routine eye examination should include measuring IOP, not only diabetic retinopathy screening. Further studies are needed to understand the mechanisms of increased IOP in DM patients beyond glycemic control.

## Figures and Tables

**Table 1:** General characteristics of the studied subjects depending on gender.

Variable	Women	Men	Difference	*p*
Gender *n* (%) ^c^	46 (52.8%)	41 (47.2%)	-	0.6
Age (years) ^a^	64.6 ± 8.6	62.4 ± 11.2	−2.1	0.3
Diabetes duration (years) ^b^	8.5 (0; 30)	10 (0; 33)	1.6	0.3
Weight (kg) ^a^	80.2 ± 15.3	93.5 ± 19.4	13.2	0.0009 *
BMI (kg/m^2^) ^a^	30.8 ± 5.7	32.3 ± 12.6	1.4	0.4
Waist (cm) ^a^	108 ± 6.2	119.6 ± 16.4	11.6	0.1
TC (mg/dL) ^a^	172.1 ± 41.5	155 ± 35.3	−17	0.09
TG (mg/dL) ^b^	143 (44; 607)	133 (48; 344)	−23.7	0.4
HDLc (mg/dL) ^b^	51 (31; 89)	42.5 (28; 71)	−6.9	0.04 *
LDLc (mg/dL) ^a^	95.3 ± 34.5	87.8 ± 33.2	−7.5	0.4
HbA1c (%) ^b^	6.9 (5.3; 12.1)	6.8 (5; 10)	−0.04	0.9
Glycemia (mg/dL) ^b^	125 (78; 252)	140 (90; 270)	10.7	0.2
Serum creatinine (mg/dL) ^b^	0.75 (0.42; 1.4)	1 (0.69; 8.8)	0.6	0.008 *
UACr (mg/g) ^b^	11.1 (2.6; 623.4)	4.9 (2.3; 150)	−67.2	0.3
eGFR (mL/min/m^2^) ^b^	80.3 (35.3; 109)	80.6 (6.5; 117.1)	−5.4	0.3
Uric acid (mg/dL)	5.4 ± 1.4	5.7 ± 1.3	0.3	0.3
GGT (u/L) ^b^	25 (9; 88)	38.3 (11; 116)	11.2	0.1
ALP (u/L) ^a^	78.7 ± 26.3	64.5 ± 23.6	−14.1	0.1
TBil (mg/dL) ^a^	0.6 ± 0.3	0.6 ± 0.3	0.0	0.9
AST (u/L) ^b^	21.5 (10; 67)	25.5 (13; 363)	11.1	0.07
ALT (u/L) ^b^	23 (9; 85)	35.5 (11; 176)	13.2	0.2
IOP right eye (mmHg) ^a^	16.4 ± 4.2	16.7 ± 4.5	0.3	0.7
IOP left eye (mmHg) ^a^	16.2 ± 4.5	16.6 ± 4	0.4	0.6
mean IOP (mmHg) ^a^	16.3 ± 4.3	16.7 ± 4.2	0.3	0.6

^a^ *T*-test; ^b^ Mann–Whitney test, ^c^ Chi-squared test; BMI = body mass index; TC = total cholesterol; TG = triglycerides; HDLc = high density lipoprotein cholesterol; LDLc = low density lipoprotein cholesterol; HbA1c = hemoglobin A1c; UACr = urinary albumin to creatinine ratio; eGFR = estimated glomerular filtration rate; GGT = gamma glutamyl transferase; ALP = alkaline phosphatase; TBil = total bilirubin; AST = aspartate transaminase; ALT = alanine transaminase; IOP = intraocular pressure; * = statistically significant *p* < 0.05.

**Table 2:** Associated comorbidities and treatment regimens in the study group.

Variable	*n* (%)
Diabetic therapies in different combinations	
Metformin	73 (83.9%)
SGLT2 inhibitor	36 (41.3%)
DPP4 inhibitor	8 (9.2%)
GLP-1 RA	32 (36.7%)
Sulfonylurea	11 (12.6%)
Basal insulin	15 (17.2%)
Intensive insulin therapy	5 (5.7%)
Statins	32 (43.8%)
Diabetes chronic complications and comorbidities	
HTN	58/76 (76.3%)
CHD	11/74 (14.9%)
Stroke	4/70 (5.4%)
CKD	13/75 (17.3%)
Neuropathy predominantly sensitive	7 (8%)
Peripheral arterial disease	4/75 (5.3%)
Eye disease	
Diabetic retinopathy	9 (10.6%)
Nonproliferative Preproliferative Proliferative	6 (6.9%) 1 (1.4%) 2 (2.3%)
Glaucoma	7/87 (8.3%)
Maculopathy	3/87 (3.5%)
Cataract	
Left eye Right eye	40/87 (45.9%) 38/87 (43.6%)
Myopia	4/87 (4.6%)
Retinal angiosclerosis	52/87 (59.8%)

SGLT2 = sodium-glucose co-transporter-2; DPP4 = dipeptidil-peptidase-4; GLP-1 RA = glucagon-like peptide-1 receptor agonist; HTN = hypertension; CHD = coronary heart disease; CKD = chronic kidney disease.

**Table 3:** Comparison of studied subjects depending on the outcome threshold for intraocular pressure.

**Variable**	**IOP < 14.5 mmHg**	**IOP ≥ 14.5 mmHg**	**Difference**	***p***
*n* (%) ^c^	27 (32.1%)	60 (68.9%)	-	0.002 *
Age (years) ^a^	65.5 ± 9.4	62.1 ± 10	−3.3	0.1
Diabetes duration (years) ^a^	11.2 ± 7.7	10.5 ± 8.8	−0.6	0.7
Weight (kg) ^a^	86.4 ± 20.4	86.6 ± 17.9	0.2	0.9
BMI (kg/m^2^) ^a^	30.7 ± 6.3	31.9 ± 10.9	1.1	0.6
Waist (cm) ^a^	106	114 ± 13.3	8	0.5
HbA1c (%) ^b^	6.8 (5.7; 10.7)	6.9 (5; 12.1)	0.1	0.7
Glycemia (mg/dL) ^a^	138.4 ± 33.6	139.9 ± 40.8	1.5	0.8
IOP right eye (mmHg) ^a^	12 ± 1.5	18.7 ± 3.5	6.7	<0.0001 *
IOP left eye (mmHg) ^a^	11.8 ± 1.8	18.7 ± 3.2	6.8	<0.0001 *
mean IOP (mmHg) ^a^	11.9 ± 1.4	18.7 ± 3.3	6.8	<0.0001 *
TC (mg/dL) ^a^	157.2 ± 32.2	166.8 ± 41.9	9.5	0.3
TG (mg/dL)	105 (59; 459)	145 (44; 607)	37.4	0.1
LDLc (mg/dL) ^a^	81.8 ± 26.1	94.9 ± 36.5	13.1	0.1
HDLc (mg/dL) ^a^	46.8 ± 17.7	47.5 ± 10.9	0.6	0.8
UACr (mg/g) ^a^	10.8 ± 7.9	76 ± 186.6	65.1	0.4
Serum creatinine (mg/dL) ^b^	0.97 (0.4; 2.5)	0.7 (0.5; 8.8)	0.1	0.6
eGFR (mL/min/m^2^) ^a^	71.8 ± 19.9	81.6 ± 25.2	9.8	0.1
Uric acid (mg/dL) ^a^	6 ± 1.6	5.4 ± 1.2	−0.6	0.1
ALP (u/L) ^a^	89.6 ± 28.5	66.7 ± 22.7	−22.9	0.03 *
GGT (u/L)	18.5 (9; 59)	38 (19.8; 116)	19.8	0.007 *
TBil (mg/dL) ^a^	0.4 ± 0.1	0.7 ± 0.3	0.2	0.02 *
ALT (u/L) ^b^	20.2 (9; 176)	33 (14; 85)	6.2	0.3
AST (u/L) ^b^	20.8 (13; 363)	26 (10; 67)	−9.3	0.4

^a^ *T*-test; ^b^ Mann–Whitney test; ^c^ Chi-squared test BMI = body mass index; TC = total cholesterol; TG = triglycerides; HDLc = high density lipoprotein cholesterol; LDLc = low density lipoprotein cholesterol; HbA1c = hemoglobin A1c; UACr = urinary albumin to creatinine ratio; eGFR = estimated glomerular filtration rate; GGT = gamma glutamyl transferase; ALP = alkaline phosphatase; TBil = total bilirubin; AST = aspartate transaminase; ALT = alanine transaminase; IOP = intraocular pressure; * = statistically significant *p* < 0.05.

**Table 4:** Multivariate regression analysis of mean IOP and factors associated in the subgroup of patients with controlled glycemia and a diabetes duration below 15 years.

	Unstandardized Coefficients		Standardized Beta Coefficient	t	*p*
	B	Std. Error			
Constant	9.07	7.29	0.00	1.24	0.22
Diabetes duration (years)	0.38	0.12	0.52	3.19	0.004
Age (years)	−0.02	0.09	−0.03	−0.17	0.86
eGFR (mL/min/m^2^)	0.07	0.03	0.38	2.06	0.05

Adjusted R^2^ = 0.29, *p* = 0.008 for overall model. eGFR estimated glomerular filtration rate.

## Data Availability

Due to the retrospective study design, the informed consent was waived; therefore, the subjects of this study did not give written consent for their data to be shared publicly, so due to the sensitive nature of the research, supporting data is not available.

## References

[1] International Diabetes Federation (2021). IDF Diabetes Atlas.

[2] Meda E., Pavkov Y.M. (2023). Diabetes and Kidney Disease. https://diabetesatlas.org/atlas/diabetes-and-kidney-disease/.

[3] The Fred Hollows Foundation and International Diabetes Federation (2015). Diabetes Eye Health: A Guide for Health Care Professionals.

[4] Dielemans I., de Jong P.T., Stolk R., Vingerling J.R., Grobbee D.E., Hofman A. (1996). Primary open-angle glaucoma, intraocular pressure, and diabetes mellitus in the general elderly population: The Rotterdam Study. Ophthalmology.

[5] Şahin A., Bayer A., Özge G., Mumcuoğlu T. (2009). Corneal biomechanical changes in diabetes mellitus and their influence on intraocular pressure measurements. Investig. Ophthalmol. Vis. Sci..

[6] Pimentel L.G.M., Gracitelli C.P.B., Da Silva L.S.C., Souza A.K.S., Prata T.S. (2015). Association between Glucose Levels and Intraocular Pressure: Pre- and Postprandial Analysis in Diabetic and Nondiabetic Patients. J. Ophthalmol..

[7] Luo X.-Y., Tan N.Y.Q., Chee M.-L., Shi Y., Tham Y.-C., Wong T.Y., Wang J.J., Cheng C.-Y. (2018). Direct and Indirect Associations Between Diabetes and Intraocular Pressure: The Singapore Epidemiology of Eye Diseases Study. Investig. Ophthalmol. Vis. Sci..

[8] VanderZee B., Shafer B.M., Berdahl J.P. (2021). Intracranial Pressure and Its Relationship to Glaucoma. Curr. Ophthalmol. Rep..

[9] Cavet M.E., Vittitow J.L., Impagnatiello F., Ongini E., Bastia E. (2014). Nitric oxide (NO): An emerging target for the treatment of glaucoma. Investig. Ophthalmol. Vis. Sci..

[10] Nakazawa T., Fukuchi T. (2020). What is glaucomatous optic neuropathy?. Jpn. J. Ophthalmol..

[11] Li Y., Mitchell W., Elze T., Zebardast N. (2021). Association Between Diabetes, Diabetic Retinopathy, and Glaucoma. Curr. Diabetes Rep..

[12] Birhanu G., Tegegne A.S. (2022). Predictors for elevation of Intraocular Pressure (IOP) on glaucoma patients; a retrospective cohort study design. BMC Ophthalmol..

[13] Faralli J.A., Filla M.S., Peters D.M. (2019). Role of Fibronectin in Primary Open Angle Glaucoma. Cells.

[14] Roberts A.L., Mavlyutov T.A., Perlmutter T.E., Curry S.M., Harris S.L., Chauhan A.K., McDowell C.M. (2020). Fibronectin extra domain A (FN-EDA) elevates intraocular pressure through Toll-like receptor 4 signaling. Sci. Rep..

[15] Sato T., Roy S. (2002). Effect of high glucose on fibronectin expression and cell proliferation in trabecular meshwork cells. Investig. Ophthalmol. Vis. Sci..

[16] Li A.-F., Tane N., Roy S. (2004). Fibronectin overexpression inhibits trabecular meshwork cell monolayer permeability. Mol. Vis..

[17] Oshitari T., Fujimoto N., Hanawa K., Adachi-Usami E. (2007). Effect of chronic hyperglycemia on intraocular pressure in patients with diabetes. Arch. Ophthalmol..

[18] Yun H., Lathrop K.L., Yang E., Sun M., Kagemann L., Fu V., Stolz D.B., Schuman J.S., Du Y. (2014). A laser-induced mouse model with long-term intraocular pressure elevation. PLoS ONE.

[19] American Diabetes Association (2021). 9. Pharmacologic Approaches to Glycemic Treatment. Diabetes Care.

[20] American Diabetes Association Professional Practice Committee (2023). Summary of Revisions: Standards of Care in Diabetes—2024. Diabetes Care.

[21] Sinha B., Ghosal S. (2021). A Target HbA1c Between 7 and 7.7% Reduces Microvascular and Macrovascular Events in T2D Regardless of Duration of Diabetes: A Meta-Analysis of Randomized Controlled Trials. Diabetes Ther..

[22] Song B.J., Aiello L.P., Pasquale L.R. (2016). Presence and Risk Factors for Glaucoma in Patients with Diabetes. Curr. Diabetes Rep..

[23] Hanyuda A., Sawada N., Yuki K., Uchino M., Ozawa Y., Sasaki M., Tsugane S. (2020). Relationships of diabetes and hyperglycaemia with in-traocular pressure in a Japanese population: The JPHC-NEXT Eye Study. Sci. Rep..

[24] Zhao D., Cho J., Kim M.H., Friedman D.S., Guallar E. (2015). Diabetes, fasting glucose, and the risk of glaucoma: A meta-analysis. Ophthalmology.

[25] Bonovas S., Peponis V., Filioussi K. (2004). Diabetes mellitus as a risk factor for primary open-angle glaucoma: A meta-analysis. Diabetes Med..

[26] Tan G.S., Wong T.Y., Fong C.W., Aung T. (2009). Diabetes, metabolic abnormalities, and glaucoma. The Singapore Malay Eye Study. Arch. Ophthalmol..

[27] Kawase K., Tomidokoro A., Araie M., Iwase A., Yamamoto T., Society J.G., Tajimi Study Group (2008). Ocular and systemic factors related to intraocular pressure in Japanese adults: The Tajimi study. Br. J. Ophthalmol..

[28] Oh S.W., Lee S., Park C., Kim D.J. (2005). Elevated intraocular pressure is associated with insulin resistance and metabolic syndrome. Diabetes/Metab. Res. Rev..

[29] McLeod S.D., West S.K., A Quigley H., Fozard J.L. (1990). A longitudinal study of the relationship between intraocular and blood pressures. Investig. Ophthalmol. Vis. Sci..

[30] Lin H.C., Stein J.D., Nan B., Childers D., Newman-Casey P.A., Thompson D.A., Richards J.E. (2015). Association of Geroprotective Effects of Met-formin and Risk of Open-Angle Glaucoma in Persons with Diabetes Mellitus. JAMA Ophthalmol..

[31] Kim Y.S., Kim M., Choi M.Y., Lee D.H., Roh G.S., Kim H.J., Kang S.S., Cho G.J., Kim S.-J., Yoo J.-M. (2017). Metformin protects against retinal cell death in diabetic mice. Biochem. Biophys. Res. Commun..

[32] Shao S.-C., Su Y.-C., Lai E.C.-C., Chang K.-C., Lee C.-N., Hung M.-J., Lai C.-C., Huang F.-C., Hung J.-H. (2022). Association between sodium glucose co-transporter 2 inhibitors and incident glaucoma in patients with type 2 diabetes: A multi-institutional cohort study in Taiwan. Diabetes Metab..

[33] Sterling J., Hua P., Dunaief J.L., Cui Q.N., VanderBeek B.L. (2023). Glucagon-like peptide 1 receptor agonist use is associated with reduced risk for glaucoma. Br. J. Ophthalmol..

[34] Hashemi H., Aghamirsalim M., Yekta A., Hashemi A., Sajadi M., Khabazkhoob M., Heydarian S. (2023). Distribution and associated factors of intraocular pressure in the older population: Tehran Geriatric Eye Study. Int. J. Ophthalmol..

[35] Khawaja A.P., Springelkamp H., Creuzot-Garcher C., Delcourt C., Hofman A., Höhn R., I Iglesias A., Wolfs R.C., Korobelnik J.-F., Silva R. (2016). Associations with intraocular pressure across Europe: The European Eye Epidemiology (E^3^) Consortium. Eur. J. Epidemiol..

[36] Chung H.J., Bin Hwang H., Lee N.Y. (2015). The Association between Primary Open-Angle Glaucoma and Blood Pressure: Two Aspects of Hypertension and Hypotension. BioMed Res. Int..

[37] Gedde S.J., Feuer W.J., Shi W., Lim K.S., Barton K., Goyal S., Ahmed I.I., Brandt J., Banitt M., Budenz D. (2018). Treatment Outcomes in the Primary Tube Versus Trabeculectomy Study after 1 Year of Follow-up. Ophthalmology.

[38] Yokomichi H., Kashiwagi K., Kitamura K., Yoda Y., Tsuji M., Mochizuki M., Sato M., Shinohara R., Mizorogi S., Suzuki K. (2016). Evaluation of the associations between changes in intraocular pressure and metabolic syndrome parameters: A retrospective cohort study in Japan. BMJ Open.

[39] Kim Y.-H., Jung S.W., Nam G.-E., Han K.D., Bok A.R., Baek S.J., Cho K.-H., Choi Y.S., Kim S.-M., Ju S.-Y. (2014). High intraocular pressure is associated with cardiometabolic risk factors in South Korean men: Korean National Health and Nutrition Examination Survey, 2008–2010. Eye.

[40] Wygnanski-Jaffe T., Bieran I., Tekes-Manova D., Morad Y., Ashkenazi I., Mezer E. (2015). Metabolic syndrome: A risk factor for high intraocular pressure in the Israeli population. Int. J. Ophthalmol..

[41] Wang S., Xu L., Jonas J.B., Wang Y.X., You Q.S., Yang H. (2012). Dyslipidemia and eye diseases in the adult chinese population: The Beijing eye study. PLoS ONE.

[42] Kang J.H., Pasquale L.R., Willett W.C., A Rosner B., Egan K.M., Faberowski N., E Hankinson S. (2004). Dietary fat consumption and primary open-angle glaucoma. Am. J. Clin. Nutr..

[43] Wang S., Bao X. (2019). Hyperlipidemia, Blood Lipid Level, and the Risk of Glaucoma: A Meta-Analysis. Investig. Ophthalmol. Vis. Sci..

[44] Wang T.-H., Tsai Y.-J., Wang Y.-H., Wu C.-L., Lin I.-C. (2023). Relationship between Dry Eye Disease and Dyslipidemia: A Systematic Review. J. Clin. Med..

[45] Lee J.-H., Kwon Y.-J., Lee H.S., Han J.H., Joung B., Kim S.J. (2022). Fatty Liver Is an Independent Risk Factor for Elevated Intraocular Pressure. Nutrients.

[46] Kwon Y.-J., Kim J.-H., Jung D.-H. (2018). Association Between Nonalcoholic Fatty Liver Disease and Intraocular Pressure in Korean Adults. Eur. J. Gastroenterol. Hepatol..

[47] Flammer J., Haefliger I.O., Orgül S., Resink T. (1999). Vascular Dysregulation: A Principal Risk Factor for Glaucomatous Damage?. J. Glaucoma.

[48] Lab Test Online Alkaline Phosphatase (ALP). https://labtestsonline.org/tests/alkaline-phosphatase-alp.

[49] Lowe D., Sanvictores T., Zubair M., John S. (2023). Alkaline Phosphatase.

[50] Makris K., Mousa C., Cavalier E. (2022). Alkaline Phosphatases: Biochemistry, Functions, and Measurement. Calcif. Tissue Int..

